# Correction to: Current and future applications of induced pluripotent stem cell-based models to study pathological proteins in neurodegenerative disorders

**DOI:** 10.1038/s41380-021-01055-8

**Published:** 2021-03-09

**Authors:** Aurélie de Rus Jacquet, Hélèna L. Denis, Francesca Cicchetti, Melanie Alpaugh

**Affiliations:** 1 Centre de Recherche du CHU de Québec - Université Laval, Axe Neurosciences, Québec, QC G1V 4G2 Canada; 2 Département de Psychiatrie & Neurosciences, Université Laval, Québec, QC G1V 0A6 Canada

**Keywords:** Neuroscience, Stem cells

Correction to: *Molecular Psychiatry*

10.1038/s41380-020-00999-7

The article “Current and future applications of induced pluripotent stem cell-based models to study pathological proteins in neurodegenerative disorders”, written by Aurélie de Rus Jacquet, Hélèna L. Denis, Francesca Cicchetti and Melanie Alpaugh, was originally published electronically on the publisher’s internet portal on 25 January 2021 without open access.

With the author(s)’ decision to opt for Open Choice the copyright of the article changed on 15 February 2021 to © The Author(s) 2021 and the article is forthwith distributed under a Creative Commons Attribution 4.0 International License, which permits use, sharing, adaptation, distribution and reproduction in any medium or format, as long as you give appropriate credit to the original author(s) and the source, provide a link to the Creative Commons licence, and indicate if changes were made. The images or other third party material in this article are included in the article’s Creative Commons licence, unless indicated otherwise in a credit line to the material. If material is not included in the article’s Creative Commons licence and your intended use is not permitted by statutory regulation or exceeds the permitted use, you will need to obtain permission directly from the copyright holder. To view a copy of this licence, visit http://creativecommons.org/licenses/by/4.0.

